# Elucidation of bioinformatic-guided high-prospect drug repositioning candidates for DMD via Swanson linking of target-focused latent knowledge from text-mined categorical metadata

**DOI:** 10.3389/fcell.2023.1226707

**Published:** 2023-08-17

**Authors:** J. Wes Ulm, Florian Barthélémy, Stanley F. Nelson

**Affiliations:** ^1^ Department of Human Genetics, David Geffen School of Medicine, University of California, Los Angeles, Los Angeles, CA, United States; ^2^ Center for Duchenne Muscular Dystrophy at UCLA, Los Angeles, CA, United States; ^3^ Department of Microbiology, Immunology and Molecular Genetics, David Geffen School of Medicine, College of Letters and Sciences, University of California, Los Angeles, Los Angeles, CA, United States; ^4^ Department of Neurology, David Geffen School of Medicine, University of California, Los Angeles, Los Angeles, CA, United States; ^5^ Department of Pathology and Laboratory Medicine, David Geffen School of Medicine, University of California, Los Angeles, Los Angeles, CA, United States

**Keywords:** data mining, drug repositioning, Swanson linking, MeSH, DMD, drug repurposing, latent knowledge, therapy

## Abstract

Duchenne Muscular Dystrophy (DMD)’s complex multi-system pathophysiology, coupled with the cost-prohibitive logistics of multi-year drug screening and follow-up, has hampered the pursuit of new therapeutic approaches. Here we conducted a systematic historical and text mining-based pilot feasibility study to explore the potential of established or previously tested drugs as prospective DMD therapeutic agents. Our approach utilized a Swanson linking-inspired method to uncover meaningful yet largely hidden deep semantic connections between pharmacologically significant DMD targets and drugs developed for unrelated diseases. Specifically, we focused on molecular target-based MeSH terms and categories as high-yield bioinformatic proxies, effectively tagging relevant literature with categorical metadata. To identify promising leads, we comprehensively assembled published reports from 2011 and sampling from subsequent years. We then determined the earliest year when distinct MeSH terms or category labels of the relevant cellular target were referenced in conjunction with the drug, as well as when the pertinent target itself was first conclusively identified as holding therapeutic value for DMD. By comparing the earliest year when the drug was identifiable as a DMD treatment candidate with that of the first actual report confirming this, we computed an Index of Delayed Discovery (IDD), which serves as a metric of Swanson-linked latent knowledge. Using these findings, we identified data from previously unlinked articles subsetted via MeSH-derived Swanson linking or from target classes within the DrugBank repository. This enabled us to identify new but untested high-prospect small-molecule candidates that are of particular interest in repurposing for DMD and warrant further investigations.

## 1 Introduction

Duchenne Muscular Dystrophy (DMD) is an X-linked, progressive degenerative muscle disease caused by a variety of mutations that disrupt the open reading frame (ORF) of *DMD*, encoding dystrophin, a critical component of the dystrophin-associated glycoprotein complex (DGC) necessary for muscle protection and repair. Symptoms related to proximal muscle weakness become evident in early childhood, progressing to loss of ambulation usually by early adolescence and fatal cardiopulmonary complications in the third decade of life (Kieny et al., 2013; Van Ruiten et al., 2016). Despite classification as a rare disease overall, DMD is among the most common inherited Mendelian conditions, manifesting in 15.9–19.5 per 100,000 live births (Mendell et al., 2012; Moat et al., 2013). Innovations in gene therapy-based modalities (dystrophin gene replacement and antisense-mediated exon-skipping) have demonstrated promise in overcoming the causative loss-of-function defect, but at present, there is no cure [for a recent review see (Barthelemy and Wein, 2018)].

Despite the inability to fully remedy the dystrophin deficiency, other therapeutic avenues have shown potential in limiting the disease’s progression (Barthelemy and Wein, 2018). Of note, although DMD as a diagnosis follows single-gene Mendelian patterns of hemizygous inheritance from carrier mothers (or from *de novo* germline mutations) in boys (Darras et al., 1993), its severity and susceptibility to intervention evince a far more complex picture, with additional genes (Bello and Pegoraro, 2019; Flanigan et al., 2023), environmental factors, and interim treatments altering disease course (Ryder et al., 2017). Moreover, the dystrophin-deficient phenotype appears to be ameliorated by various mitigating factors even in untreated patients, such as sporadic revertant fibers (Ryder, Leadley, Armstrong, Westwood, de Kock, Butt, Jain and Kleijnen, 2017) and partial complementation of dystrophin loss via redundant mechanisms in the dystrophin-associated glycoprotein complex as well as potentially through utrophin, a cytoskeletal homologue of dystrophin upregulated in DMD (Kleopa et al., 2006). Notably, studies have suggested that macroscopic muscle function can be significantly preserved even if only a modest fraction of fibers retain functional DGC’s, either through partial dystrophin restoration or compensatory mechanisms (Tinsley et al., 1996; Tinsley et al., 1998; Gilbert et al., 1999; Kleopa, Drousiotou, Mavrikiou, Ormiston and Kyriakides, 2006; Johnson et al., 2013). Thus DMD’s phenotypic presentation in any given patient stems from a more intricate and multigenic picture with epistatic effects.

Consequently, even in the absence of dystrophin restoration, there are fruitful therapeutic avenues to boost muscle function and reduce disease burden. Coupled with the increasing cost-effectiveness of whole exome and partial or full genome sequencing, such methods may help customize DMD treatment regimens through tailored pharmaceutical interventions for each patient. Findings from a number of studies, such as the survey-based Duchenne Registry, have likewise indicated that readily available drugs and supplements can bolster patient outcomes alongside traditional corticosteroid regimens (Wang et al., 2014). Additionally, some pilot investigations have shown that combination therapies incorporating long-established drugs can engender marked improvements in DMD patients’ health (Cordova et al., 2018).

Despite the potential benefits of cost reductions and leveraging proven therapeutic interventions in drug repositioning, systematic drug repurposing for neuromuscular disease (NM) remains limited in outcome data. This is evident both generally and in specific studies such as those conducted by Cha et al. (2018) and Clout et al. (2019). Particularly promising examples of such repurposed agents in the realm of NM disease management include the free radical scavenger edaravone for amyotrophic lateral sclerosis (Aoki et al., 2011; Takei et al., 2017; Shah et al., 2021), metformin for Steinert myotonic dystrophy (DM1) (Bassez et al., 2018; Gutiérrez-Gutiérrez and Rosado-Bartolomé, 2020), and omaveloxolone and deferiprone for Friedreich’s ataxia (Lynch et al., 2021; Shah, Dooms, Amaral-Garcia and Igoillo-Esteve, 2021), all of which have demonstrated some clinical improvement in individuals or patient cohorts. Additionally, several recent reports have indicated enhanced potential of selected repurposed therapies in animal or tissue-derived cellular models for significant neuromuscular disorders, for example metformin in BAG3 myofibrillar myopathy models (Ruparelia et al., 2021), moxifloxacin (Januel et al., 2022) and VPA + PMO combination therapy (and other varied PMO-combination modalities) for SMA (Farrelly-Rosch et al., 2017; Richelme, 2019), and the antibiotic florfenicol for Charcot-Marie-Tooth (CMT) disease (Nuevo-Tapioles et al., 2021). Although several classes and instances of repurposed drugs have been examined for potential against DMD, including metformin, tamoxifen, simvastatin, gentamicin, and tadalafil (Vitiello et al., 2019), most are in pre-clinical stages or have not demonstrated significant clinical improvement. However, some such as tamoxifen, have shown more promise in ameliorating the clinical phenotypes in varied patient cohorts (Tsabari et al., 2021; Guglieri et al., 2022).

Interest has therefore mounted in the potential of drug repurposing as a rapid, efficient, cost-effective, and readily available path to treat DMD patients and attenuate disease phenotype. The prospect of repositioning existing therapeutics, particularly drawing from a vast trove of small-molecule agents already characterized, also holds the promise of more rapidly bringing efficacious interventions into the clinic, obviating many of the long delays and prohibitive costs that hamper testing and implementation of entirely novel agents. Nevertheless, screening of DMD drug candidates is hindered by the complexity of its chronic pathophysiology both at the cellular and tissue level, and the consequent hurdles of establishing proper metrics to identify genuine hits with likelihood of success for *in vivo* and clinical trial testing. Therefore, informatically-guided pre-selection of likely candidates may greatly facilitate such searches, as undertaken recently in drug repositioning for other conditions, notably including COVID-19 (Ulm and Nelson 2021).

We conducted a pilot feasibility study on a Swanson linking and text mining-based approach to find potential candidates for DMD drug repurposing. This will serve as a foundation for further research on deep semantic structures and natural language processing to connect overlooked disease targets with available treatments or drug classes. Text mining-guided drug candidate screens in general must tackle the core informatic challenge of programmatically distilling large, highly variable data sources into simplified summary statistics without sacrificing essential lexical information. Systematic analysis of text-based data sets and comparative textual analysis face the challenge of balancing algorithmic manageability and Kolmogorov complexity. For purposes here, this complexity metric essentially measures the irreducible critical information needed to meaningfully describe a promising drug prospect, its/their mechanism(s), molecular target(s), and the implicit categorical descriptors that can actionably link these characteristics to other drugs and targets.

To circumvent this obstacle and develop a proof-of-concept to yield actionable information, we have taken advantage of DMD’s evolving history as a focal point of diverse modalities–both in animal models and in human studies and clinical trials–focusing on National Library of Medicine Medical Subject Headings (MeSH) as a bioinformatic proxy to signal meaningful semantic and categorical links. It is these hidden but significant semantic connections that are of interest to methodologies variously classified as Swanson linking, an investigational tool to uncover latent knowledge in a form accessible to programmatic analysis and follow-up. These approaches are detailed here.

## 2 Methods

DMD-related research reports were initially scraped in the period from 1970 through 1986—the year of dystrophin’s positional cloning–using PubMed direct-search and programmatic (NCBI E-utilities) approaches to acquire a sampling of articles following up on DMD drug prospects. The available literature was then divided into 4-year inclusive blocks, from 1970 through 2017, to provide for partitioned queries returning the number of distinct reports mentioning each drug prospect and respective target, both before and after the positional cloning. We concomitantly generated comparative alphabetically-ordered word clouds for each decade from 1970 (and for the years 2020–2023), based on tokenized abstract-comprised corpora for each period, to visualize aggregate macroscopic trends and shifts in research focus of DMD treatment-related reports over the past half-century (Figure 2). Following this initial historical survey, we proceeded with the principal analysis (summarized in Supplementary Table S1): ascertaining the earliest possible timepoints of data-minable deep semantic linkages for drugs tested for efficacy against DMD in animal models and human trials.

**FIGURE 1:**
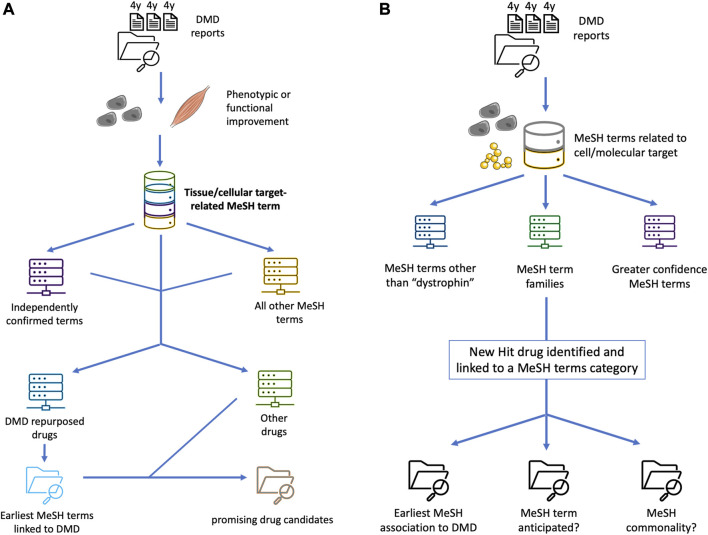
Conceptual schematics of enhanced applied Swanson linking for DMD drug repurposing, diversifying the data buckets to help expand the pool of prospects and better pinpoint promising treatment candidates. **(A)** schematizes an additional step of using previously repurposed or repurposable drugs in an essentially recursive process, to expand the pool of MeSH terms or other bioinformatic markers of latent knowledge and significant semantic linkage, based on e.g. an unanticipated nexus with a cellular pathway, upstream or downstream epistasis, or systemic physiological effects. **(B)** shows a “siloing” of different MeSH-based Swanson links based on priority and a prospective scoring system, for example based on independent confirmation of a strong drug-target interaction by multiple groups, high efficacy in several animal models, higher demonstrated relevance for DMD pathophysiology, quantification of agonist-like or antagonist-like effect, or data available from full clinical trials undertaken for other diseases.

Focusing initially on 2011 for a systematic examination of tested pharmacologic agents–both newly discovered and previously known–we then identified a sample of additional novel drug prospects in succeeding years, of interest particularly due to subsequent clinical follow-up and/or novel pharmacologic classes, reported with experimental confirmation of potential efficacy. For each drug with known pharmacodynamics and mechanism, a principal target or targets in the catalogued report were noted and MeSH terms and term categories (directly included or gleaned from title or keyword listings) were gathered as an atomic categorical data element for Swanson linking (diagrammed, in its broad conceptual approach, in Figure 1). Columnar data for each case was then provided in categorical, quantitative, or ASCII string format in the following core domains: first author, PMID, publication year (if after 2011), first reported year of the drug’s association with its listed target or target class plus associated PMID of that report, the first year in which that target’s clinical significance for DMD was established (and that report’s PMID), the Discovery Year 0 (later of the 2 years, when the drug could first be semantically and algorithmically Swanson-linked as a DMD drug prospect), the year and PMID of the first report demonstrating the drug’s prospective efficacy for DMD, and the Index of Delayed Discovery (IDD) calculated as the difference between that report’s year and Discovery Year 0. In cases for which the printed article appeared following an electronic publication, the earlier of the 2 years (if applicable) was taken as the publication date. In the event of drug combinations, the earliest year of significance as a DMD drug target was taken as the first year in which *both* drugs’ relevant targets were found to be germane to DMD pathophysiology (i.e. the later of the two).

Additional columns were then filled with primarily categorical data elements that could function as informatic proxies for the underlying deep semantic Swanson links, thereby serving as interrogable and minable bridges for the determination of additional high-prospect DMD drug candidates. These include a summary descriptor to classify the drug’s action relative to its target(s) as agonist-like, antagonist-like, or neither; pertinent primary and secondary drug/target-related MeSH terms; a categorical classifier of the drug candidate’s validation (whether it did or did not ameliorate the DMD phenotype in the report); abstract-derived semantic outcome markers as single-word instances, bigram instances (distinct morphemes represented as set two-word pairs), and instances of trigrams or above; a categorical classifier of the model organism (or confirmation of a clinical trial); and the clinical trial record number if relevant. For DMD pharmacologic targets identified by especially promising drug candidates with knowledge of their pharmacodynamic effects and pathways, these rows were then used to construct queries of NLM databases and the DrugBank target database to elicit additional high-prospect drug candidates (Wishart et al., 2006). A sampling of such findings is furnished in Supplemental Table S3, as detailed below.

## 3 Results

This pilot feasibility study was motivated by renewed interest in small-molecule pharmacotherapies that relieve DMD phenotype without widespread myofiber dystrophin restoration. This interest has grown gradually over the past two decades. Studies of such drug prospects had been commonplace particularly in the 1970s and early 1980s, prior to the causal genetic knowledge, but declined in frequency after dystrophin’s positional cloning, which created an entirely new paradigm to consider pathologic disruptions and therapeutic targets. With a need for significant therapeutic gains in various domains of DMD management and limitations in practical experimental options, it is valuable to have a systematic understanding of the intricate history referenced above. This can aid in identifying new or previously disregarded therapeutic choices that may enhance existing dystrophin restoration therapies, either as monotherapies or in unexplored combinations. We therefore commenced with a historical overview and retrospective analysis of treatment candidates under examination since 1970. Some were first tested in the early 1970s, some followed after the dystrophin ORF was identified in 1986 and gene therapeutic modalities were being explored; but in each case, the drug candidates of interest were largely dropped without further examination or explicit rule-outs in clinical studies.

**FIGURE 2:**
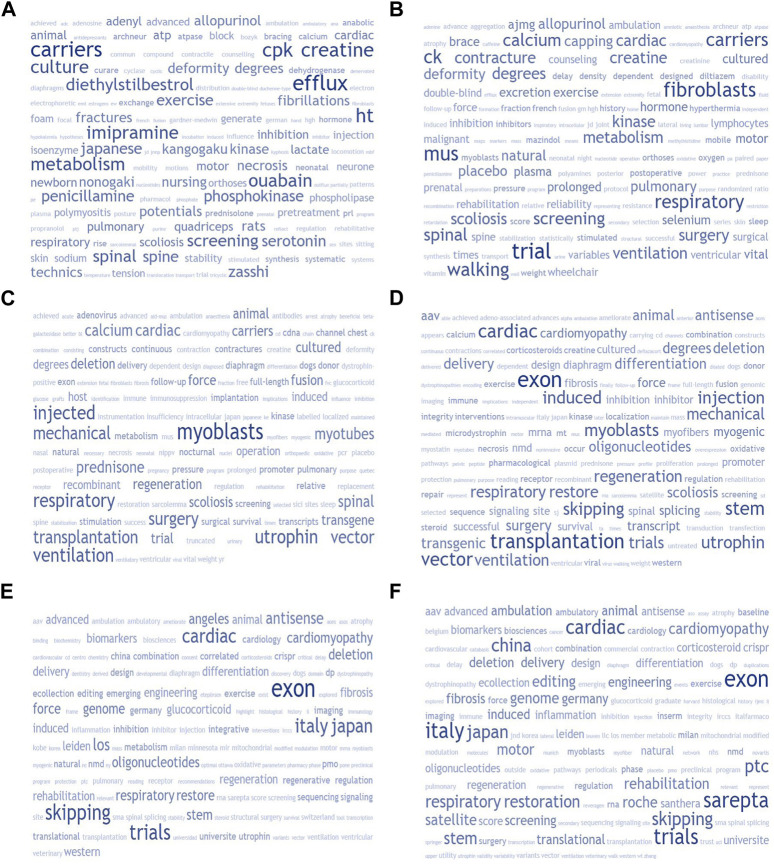
The study used tokenized term frequency-based word clouds based on semantic relevance to compare trends in research focus on DMD therapeutics investigations on a decade-by-decade basis. The study started with 1970 and used corpora derived from aggregated abstracts for each period. The Entrez E-utilities API of the National Center for Biotechnology Information (NCBI) was used to generate each corpus. The ESearch PubMed database query with standardized keyword-based treatment-focused search terms was kept consistent across all periods. The study used a customized stop words list of 2,303 terms to remove “noise tokens” that would crowd out the signal of interest. The resultant 150-token word clouds provide a bird’s-eye view of the chronological evolution of DMD therapeutics-related research focus.“. We generated six panels, each representing a 10-year block: 1970’s **(A)**, 1980’s **(B)**, 1990’s **(C)**, 2000’s **(D)**, 2010’s **(E)**, with the exception of the last panel **(F)** which described the past 3 years (2020–2023).

Supplementary Table S2 provides a historical overview sampling such “non-canonical” DMD drug candidates, with a tally of the total number of DMD-related publications catalogued over 4-year blocks, and the mean number of appearances per year over the 1970–2017 stretch. All of these compounds demonstrated a broadly similar pattern, attracting a brief spell of interest (notably gentamicin, allopurinol and pentoxyfilline) and examination before largely disappearing from the scene as gene replacement and myofiber restoration strategies became predominant. We also applied the same quantification to diverse putative drug targets in the treatment of DMD outside of myofiber restoration (with calcium, utrophin, and fibrosis being the main actionable targets–still pursued to this day). In each case, a prospective molecular target was elucidated generally long before the variable phenotypic expressivity involving dystrophin frameshift mutations–and potential for partial repair and clinical improvement–was understood. From there, we used a programmatic aggregate query protocol to create a corpus of DMD therapeutics-related abstracts for each decade from 1970 (and for 2020–2023), filtered with a tailored stop words list to generate comparative word clouds for each period, shown in Figure 2. This allowed us to highlight that in the past few decades, there has been a shift in focus in the field of muscular dystrophy research. Initially, attention was on intercellular pathways, small molecule drugs, and surgery/rehabilitation methods. However, gene therapy, recombinant DNA, and utrophin-based investigations became more prevalent in the 90s–2000s. This then transitioned to gene-editing and exon-skipping approaches in the 2010s. Corticosteroid-based explorations have remained prominent throughout all time periods, while fibrosis, anti-inflammatory drugs, oxidative damage mitigation, stem cells, and biomarkers have also been areas of interest. Deflazacort has emerged as an alternative to standard prednisone protocols emerging by the 2000s.

Following the historical survey, we used Swanson-linking approaches to identify drug candidates suitable for DMD treatment regimens. We focused on MeSH terms, subheadings, and broader categories to establish deeper semantic relationships between reports, and tested whether MeSH term collections could anticipate discoveries of drugs with potential efficacy against DMD. We chose a range of drugs tested predominantly in animal studies in a recent year to ascertain deep semantic linkages of target-related MeSH assignments, and compared the earliest year of such linkages for the common DMD-pertinent target(s) to the drug (often first registered in unrelated therapeutic studies for other disorders), to determine the Discovery Year 0 for each intervention. We then computed the Index of Delayed Discovery (IDD) to quantify and retrospectively validate the latent knowledge for prospective drug repositioning. From there, we acquired further data for each drug in several domains as a foundation for information useable in prospective query engines or artificial intelligence training sets. We then summarized the drug’s mode of action as agonist-like or antagonist-like where possible.

For our pilot analysis here, there were a total of 27 DMD therapeutic agents identified from a comprehensive sweep of reports for 2011, and 38 total modalities including samples from subsequent years. Many in the first group had an IDD value of zero, signifying that 2011 was the first year during which a drug’s target was matched to a target relevant for DMD pathophysiology. The highest IDD value for both the 2011 drugs and those examined subsequently, spanning 27 years, was determined to be for ascorbic acid, which was found to be protective against oxidative damage for diaphragmatic muscle in the mdx mouse model. The mean IDD and standard deviation for the 2011 drugs was 8.28 ± 9.56 years, and 6.56 ± 8.51 years for all 38 sampled drugs taken together, indicating significant variation in latent knowledge for the various interventions and when this knowledge was applied in practice.

The median IDD value for the 2011 modalities was 6 years, and 3.5 years when including the sampled drugs from subsequent years. The mode of IDD values for the 2011 modalities, for cases in which a drug’s potential for DMD had been anticipated, was 12 years, and 7 years when including the drugs sampled in the following years. As regards the nature of the listed investigations, among the 2011 studies indicating demonstrable interventional efficacy (23 of the 27), 4 were clinical trials, 2 involved case reports, and the rest (17) were animal models–with one of the effective drugs tested in a mouse model being utilized in a later clinical trial as of the current date. Another clinical trial and a comparative study produced mixed results. (ACE inhibitors have been assayed in clinical studies for cardioprotective effects, though not for assessment of fibrosis reduction in skeletal muscle *per se*.) Of the post-2011 studies, one clinical trial produced mixed results, while 10 showed efficacy in animal models (mouse and dog) of which 6 were followed by subsequent clinical studies, several in progress.

We then applied our framework to future prospects for DMD drug repurposing studies, selecting target-related MeSH terms (individually or in Boolean statements) with semantic linkages to studies with promising therapeutic candidates from prior investigations. As shown in Supplementary Table S3, our results demonstrated numerous untested therapeutic prospects identified through MeSH-term Swanson linking. These prospects showed efficacy against targets that had previously been confirmed to ameliorate the DMD phenotype (from 2011 and other years sampled) and exhibited both agonist-like and antagonist-like activity (Wishart et al., 2006). A parallel exploration was conducted on the DrugBank database, using designations equivalent to the target-relevant MeSH terms on the left side of the table. Since the DrugBank hits are typically more advanced in the development and authorization pipeline, we placed particular emphasis on drug candidates that had already been approved by centralized regulatory agencies, as well as prospective treatments (both approved for other purposes and still in investigative or experimental phases) with demonstrated empirical effectiveness against the target of interest.

Remarkably, even with this relatively small sample, dozens of new promising prospects were identified. For instance, we have identified 9 new potential ACEi/ARBs showing promising bioavailability, better half-life or the potential to be used in combination with other antihypertensive medications. Interestingly, Cyt006-Angqb is a vaccine, offering a new mechanism of action and an easier delivery method (one injection lasts weeks) in comparison to the current daily oral administration, with the potential to ameliorate compliance. Histone Deacetylase Inhibitors (HDAC) are also under active investigation (Consalvi et al., 2014; Spreafico et al., 2021) and while a few of the newly identified compound present a minor liver injury risk, some look particularly appealing due to their ability to be used in combination with other drugs (Panobinostat, Vorinostat, Decitabine). Interestingly, valproic acid has been identified as a skipping booster drug in mdx mice, offering another potential use as a combinatorial therapy, similar to dantrolene as previously published (Kendall et al., 2012; Barthelemy et al., 2019).

## 4 Discussion

In this study, Swanson linking was used to explore deep semantic relationships for drug targets in Duchenne Muscular Dystrophy. This led to a framework for expedited drug repositioning based on the latent knowledge uncovered. Target-related MeSH terms, subheadings, and categories were used as categorical data elements to specify the deep semantic links. A means to quantify the latent knowledge was provided using an Index of Delayed Discovery. This metric compares the first year of a repurposable drug’s application to DMD therapy relative to the first year that Swanson linking would have demonstrated an intersection of the drug’s target profile with the parallel target profile of DMD’s underlying pathophysiology. The mean and median values of an IDD will of course vary depending on the specific sample of drugs chosen (as many of the post-2011 drug candidates were fairly novel agents with recently described pharmacodynamic mechanisms). Nevertheless, it is remarkable that so many of the therapies found to be potentially efficacious for DMD in 2011—for which a more systematic sweep of tested drugs was undertaken–were effectively data-minable as repositioning candidates based on Swanson linking of their intersecting target profiles from many years before.

It is therefore encouraging that the deep semantic ties of even a pilot study with a Swanson linking protocol can yield such a vast wealth of newly repurposable drugs linked to well-characterized targets (as well as recapitulating treatments previously identified as promising, as shown in Supplementary Table S3), of highly varied chemical classes and diverse mechanisms that may prove germane in as-yet unrealized ways to engendering clinically significant improvements. For instance, Swanson linking on histone deacetylase inhibitors revealed novel prospects from synthetic chemical classes verified in unrelated studies [e.g., 2-aminobenzamides and quinazolinyl-containing benzamide derivatives, (Zhang et al., 2021; Bülbül et al., 2022)] or well-established agents long available as antimicrobials [e.g., trichostatin A, (He et al., 2011)]. Drug candidates displaying a nexus with TGF-beta and related pathways as a target ranged from a variety of small molecules to neutralizing antibodies [1D11, (Li and Pan, 2021)] to nutraceuticals [capsaicin, (Sugiyama et al., 2021)].

There are several salient limitations to note here, particularly for a pilot feasibility study of this type. As indicated before, there is a constant tension between preserving the intricacy of the underlying biology and distilling it into an algorithmically manageable form, and there are some cases where this balance is especially difficult to strike. This can be seen even in fairly well-characterized drug classes, e.g., with urocortins and corticosteroids–a class of mainstay drugs for DMD–which entail intricate pharmacodynamics that cannot be easily classified as “agonist” or “antagonist”, and therefore elude categorization under categorical data variables or programmatic approaches. It is similarly difficult to examine potential modalities involving synergistic administration of repurposed drugs, which–for example through dovetailing antagonism of fibrosis with agonism of utrophin production–may be among the most promising drug repositioning prospects.

The descriptors of semantic relationships for relevant drug targets can contain a certain degree of imprecision, which can present challenges in accurately predicting repurposable drugs. This is exemplified by the utilization of “scalars” in Supplementary Table S1 to express meaningful associations among drugs, disorders, and targets without specifying the direction of an effect. For example, whether a drug exerts an agonist-like or antagonist-like activity is described as a “vector” without precision. Such imprecision is also evident in the descriptors for DMD drugs that modulate the renin-angiotensin system (e.g., ACE blockade and ARBs), where their potential as high-prospect drug candidates is linked to more recent discoveries of their effect on skeletal muscle pathophysiology, beyond their initial indications as components of standard treatment cocktails for DMD-associated cardiomyopathy.

In essence, even the most rigorous programmatic approaches to the complex phenomenon of systems biology and emergent properties are inevitably characterized by a degree of irreducible arbitrariness and conjecture. As such, the biological whole will always surpass the sum of its parts, making it a challenge to find the proverbial “sweet spot” between actionable information depth and algorithmic malleability in text-mining, AI training, and data-mining. This challenge is particularly acute in the case of therapy-target interactions significant for drug repositioning and multisystem diseases such as DMD, which are characterized by complex clinical manifestations. It is important to note that a degree of data loss and imprecision will always be present in the semiotic representation of deep semantic relationships that define pathophysiology and guide treatments. This applies not only to the translation of complex cell and tissue biology into relevant texts but also to the “lossy compression” of these texts into structured data that can be used to output actionable signals through systematic queries.

Despite these limitations, the potential for even rudimentary Swanson linking to reveal otherwise unrecognized treatment candidates is quite striking. However, it is worth noting that this study’s approach to identifying latent knowledge lacks sophistication compared to more complex methods of establishing semantic links. Indeed, another salient challenge for such a pilot study is that, as seen in the outcome tabulations, it will shine a light upon substantially more promising therapeutic candidates than can be cost-effectively screened via follow-up *in vivo* investigations. Outside of preliminary *in vitro* (cell and tissue-level) testing to confirm core pharmacodynamic activity, it would be helpful to fine-tune the high-prospect candidates through a more specific, quantifiable ranking system, based on both programmatic and curated inputs rooted in the very latent knowledge uncovered by the deep semantic linkages as discussed. Such expanded methods, also illustrated in Figure 1, might include detailed semantic maps linking target-descriptive MeSH terms to additional relevant but initially undetected terms. By doing so, other elements of the target’s cellular and intercellular pathways or systemic physiology could be depicted, or a “scoring” system could be added to a MeSH term or grouping based on rigorous indices of therapeutic prospects.

The potential for deeper-layered Swanson linking is exemplified in the case of tamoxifen, an estrogen modulator that has been used to treat or prevent breast cancer, but was also found to have protein kinase C antagonism and thus could be used to treat bipolar disorder, as well as other neuropsychiatric conditions (Palacios et al., 2019). Hints of such pathway-spanning implicit semantic connections can likewise be seen in Supplementary Tables S1, S3, for example with a variety of drugs that have, directly or indirectly, shown efficacy across multiple target classes–such as inhibitors of muscle damage from NF-kappa B and TLR4 activity (Cirmi et al., 2022; Feng et al., 2022; Yang et al., 2022), first implied by an otherwise serendipitous observation involving a licorice component, glycyrrhizin, and its antagonism of toll-like receptor activity (Li et al., 2008). In any case, the framework demonstrated in this pilot study can be instrumental in providing a foundation for such advanced protocols for establishing deeper semantic relationships and eliciting actionable latent knowledge, particularly given the wealth of repurposable drug candidates already in evidence from core approaches alone.

## Data Availability

The raw data supporting the conclusion of this article will be made available by the authors, without undue reservation.

## Supplementary material

SUPPLEMENTARY TABLE S1 Drugs or drug combinations directly tested for efficacy as a treatment for DMD in human trials or animal models, from comprehensive sweep of 2011 investigations (upper part) and a selection of interventions from several categories in subsequent years (“studies after 2011”), used to ascertain the Index of Delayed Discovery and relevant MeSH term and target markers for Swanson linking. The columns provide descriptive information and metadata pertinent to the study of interest and to its potential links to latent knowledge on other DMD drug prospects, including “MeSH text mining (year)” which defines the first reported year when the target was mentioned in association with the drug; “Clinical significance for DMD established (year)” which defines the first year when the target and relevant MeSH term markers were associated with DMD; “Discovery year 0” which denotes the first year when the drug or combination was rationally repurposable as a DMD therapy via Swanson linking; “Proven efficacy for DMD” which expresses the first year when the drug was found to be efficacious as a DMD treatment (either on or before the year of the study in each row); “Index of Delayed Discovery” which helps to encapsulate the latent knowledge from Swanson linking and represents the chronological gap in years between potential and actual repurposing with histogram comparing the IDD between drugs for drugs studied before 2011 or after 2011; Graphical representation of the IDD where the first column represent the baseline of discovery year 0 and the second histogram represents the first year of proven efficacy; specific MeSH terms, subheadings, or categories applicable for further drug repositioning; and additional fields (such as outcome markers, agonist-like or antagonist-like mode of action, success or failure of the testing, animal model, and clinical trials if pertinent) that help further characterize the drug’s pharmacodynamics and efficacy, and provide additional information in several data types for programmatic AI training or query engine construction and design.

SUPPLEMENTARY TABLE S2 Normalized historical frequency in 4-year blocks of "rarely tested" drugs for DMD in blue and of DMD drug targets from before dystrophin cloning in black (number of citations/total DMD abstracts in block * 1000 in black). Each case is color-coded based on their frequency with a scale from red (value of zero up to green for the higher values). The mean column represents the ratings from 0 to 5 for all the drugs based on their frequency in comparison to the others. Proteins involved in muscle structural maintenance are highlighted in yellow; proteins involved in protein degradation and turnover are highlighted in orange; proteins regulating homeostasis through signaling pathways are highlighted in green ; proteins regulating muscle growth or muscle mass are highlighted in blue; Antioxidants are highlighted in gray; all other mechanisms of action are highlighted in pink. Inhibitors are written in purple. For each drug, their main roles in muscle, their link to DMD and the main PMID of interest are indicated.

SUPPLEMENTARY TABLE S3 High-prospect novel DMD drug repurposing candidates based on targets of verified significance linked to relevant drugs via NLM resource text-mining. Using target specifications based on MeSH and equivalent DrugBank descriptions, classed where possible as agonist-like or antagonist-like, the chart lists promising drug candidates for investigation as DMD therapeutics, as indicated by MeSH-based hits and within the DrugBank database. A single asterisk after a drug (from the DrugBank listing) indicates that the drug has already demonstrated a relevant pharmacological effect, while a double-asterisk indicates that the drug has already been tested, and shown some measurable effect, in ameliorating the DMD phenotype in an animal model, human patients, or both (and is not the drug which helped identify the MeSH classification to begin with). Additional candidates identified in the DrugBank database are indicated with their mechanism of action (if known), their current indications/investigations, their potential advantages/disadavnatges in comparison to current drugs and a few PMID of interest. Small moleculaes are written in black, protein-based therapy are indicated in green. Investigational drugs are highlighted in gray whereas approved drugs are not highlighted.
